# An appraisal: how notifiable infectious diseases are reported by Hungarian family physicians

**DOI:** 10.1186/s12879-018-2948-5

**Published:** 2018-01-17

**Authors:** Katalin Vraukó, Zoltán Jancsó, László Kalabay, Andrea Lukács, Gabriella Maráczi, Lajos Mester, Anna Nánási, József Rinfel, Tamás Sárosi, Ferenc Tamás, Albert Varga, József Vitrai, Imre Rurik

**Affiliations:** 1 Faculty of Public Health, Department Family and Occupational Medicine, University of Debrecen, Debrecen, Hungary; 2 Faculty of Medicine, Department of Family Medicine, Semmelweis University, Budapest, Hungary; 3 Faculty of Medicine, Department of Dermatology, Semmelweis University, Budapest, Hungary; 4 Government Office for Békés County, Department of Public Health, Békéscsaba, Hungary; 5 Faculty of Medicine, Department Family Medicine, University of Szeged, Szeged, Hungary; 6 Institute of Primary Care, University of Pécs, Pécs, Hungary; 7 National Institute for Health Development, Budapest, Hungary

**Keywords:** Family physician, Hungary, Infections, Infectious diseases, Primary care, Reporting

## Abstract

**Background:**

Within the frame of National Epidemiological Surveillance System, family physicians have an obligation to report infections and suspicions cases.

The aim of this study was to evaluate the knowledge, attitudes, daily practice and the reporting activities of Hungarian family physicians regarding to infectious diseases.

**Methods:**

A self-administered survey was developed, validated and used. The survey was completed by family physicians who had taken part in continuous medical educational programmes of all Hungarian medical faculties. The questionnaire, consisting demographic questions and 10 statements about their reporting habits were completed by 347 doctors, 8% of the total number of family physicians. The data were processed in a cross-sectional design with general linear model.

**Results:**

According to the majority of responders, the current reporting system works efficiently. Rural physicians were mainly agreed, that reporting is not a simply obligation, it is a professional task as well. They were less hindered in daily work by reporting activities, waited less for laboratory confirmation before reporting, reported suspicious cases more frequently.

Practitioner’s based in urban settlements preferred to await laboratory tests before reporting and were hindered less by failures of the electronic reporting system.

Older physicians trusted more in the recent system and they wished to increase the number of reports.

Female physicians have higher consciousness in epidemiology. They were mostly in agreement that even severe infectious diseases can be diagnosed at primary care level and their daily practices were less burdened by reporting duties.

**Conclusions:**

Both the epidemiological knowledge of general practitioners’ and the electronic surveillance systems should be improved. There is a need to develope the electronic infrastructure of primary care. More and regular control is also expected by the health care authorities, beside the synthesis of professional and governmental expectations and regulations.

**Electronic supplementary material:**

The online version of this article (10.1186/s12879-018-2948-5) contains supplementary material, which is available to authorized users.

## Background

Epidemiological surveillance entails the continuous and systematic collection, analysis, understanding and dissemination of health care data. Special attention is given to the incidence of infections with respect to the time and the geographic area. Furthermore, risk factors are analysed in order to take adequate preventive and restrictive measures [1–4]. Reporting the occurrence or the suspicion of infectious diseases to health authorities is an obligation not only in Hungary but in any EU member states, stipulated by law and regulation [5–8]. The initial patient-doctor doctor consultation takes place most frequently in primary care, thus this is the most important site for epidemiological surveillance [2, 9]. The results of international studies show that, despite the obligations family physicians do not always forward the epidemiological information to the health authorities [7–14]. In Hungary, all practising physicians are obliged to report and keep a record of infected and potentially infected patients based on case definitions by Additional file 1 [15]. These cases should be notified to the local health authority as well. Healthcare professionals are also obliged to inform the authorities about the outcomes of the disease if the patient, suffering from the reported condition, had complications, developed a long-term organic malformation or died.

The aim of this study was to explore the knowledge, the habits and the daily practice of Hungarian family physicians with respect to the reporting of infectious diseases to the primary care surveillance system.

## Methods

To answer our research questions a survey was run involving family physicians attending continuous medical education (CME) programs organised by the four medical faculties in Hungary. The participants were recruited during their CME program and were asked to fill in a questionnaire. This cross-sectional study was carried out in the last quarter of 2015, covering the whole country. The questionnaire was developed based on a former longitudinal study including demographic questions and 10 statements about the reporting habits of family physicians related to infectious cases (Additional file 2) [11]. The validated questionnaire was previously tested by an expert group comprised of representatives of the four Hungarian Departments of Family Medicine [16].

Participation was voluntary, a participation rate of about 80% was achieved, and answers were processed anonymously.

Our research questions were:

- How could the current epidemiological surveillance system be improved with the insights of family physicians?
- What information do family physicians have on the reporting system of infections?
- Is there any correlation between the gender, age, previous experience, geographical location of practices and their reporting habits?

The data assessing demographic characteristics were: gender, age, location and years spent in practice. Physicians were asked to rate their answer options between 0 = fully disagree, 10 = fully agree.

For the statistical analysis, the *Stata 11* software was used. The descriptive statistics were produced by the ‘summarize’ command and the regression analysis was performed using the ‘glm’ command. The association between dependent variable (agreement score on statement) and independent variables (gender, age, practice time, practice location) was analyzed using a general linear model (with maximum likelihood optimization). In this model the regression coefficient presented for categorical variables (gender, practice location) means one unit increment or decrement of score (respectively to positive or negative sign) compared to the reference category. The reference category for the gender was ‘male’ and for the practice location ‘the capital’.

In Hungary, researches in the field of medicine, human studies and their ethical issues are regulated by the Governmental Decree of 235/2009. Neither this regulation, nor other is dealing with questioning, sampling of opinion of health care providers. This means, that no ethical permission is required for this type of studies/surveys.

## Results

The survey was completed by 347 (186 female and 161 male) family physicians; their age was 54.6 ± 11.1 (mean ± SD). The years spent in practice varied between 1 to 55 (21.9 ± 11.9, mean ± SD). The doctors evaluated the statements in Table 1. The association between statements and demographic characteristics are presented in Tables 2 and 3.

**Table 1:** Summary of agreement scores given to the statements on reporting system of communicable diseases

Statements	Mean score	Percentile 25th	Percentile 75th
Infections reported by one single doctor do not contribute to epidemiological surveillance	3.16	0	5
Reporting infectious diseases hinders daily work	3.39	0	6
I cannot report due to system failure (system not available) from time to time	4.89	1	8
Only lab proven infectious diseases will be reported	5.67	2	9
Reporting infections takes more time that they are usually short of	6.09	3	9
Only serious infections should be reported	6.19	4	9
Severe infections are detected in special care	6.22	5	8
The national infectious disease surveillance system works well	6.86	5	8
It is not clear how reporting data will be used by health care authorities	6.79	5	10
Reporting is not only an obligation stipulated by law but also a professional task	9.03	9	10

Percentile 25th and Percentile 75th marks the scores under which one third and three third of scores were given, respectively

**Table 2:** Association between agreement scores and gender, age, years spent in practice

	Statements	Gender		Age		Years of practice	
		Coef.	p	Coef.	p	Coef.	p
1.	The Hungarian infection surveillance system works well.	0,36	0,239	−0,13	0,564	**0,04**	**0,018**
2.	Severe infectious diseases are detected in special care.	−0,55	0,098	−0,16	0,519	0,01	0,951
3.	I only report lab proven infection cases.	−0,18	0,704	0,03	0,369	0,02	0,454
4.	Infection cases exclusively reported by one single doctor do not contribute to epidemiology surveillance.	−0,41	0,381	**0,07**	**0,034**	0,09	0,766
5.	Reporting infections cases hinders my daily clinical work.	−0,79	0,076	0,04	0,184	0,03	0,211
6.	Reporting infection cases is not only an obligation by law but is also a professional task as well.	0,31	0,209	0,05	0,991	0,06	0,707
7.	Only relevant and severe infection cases should be reported.	−0,66	0,154	0,01	0,973	0,07	0,814
8.	Reporting infections requires more time that we are sometimes short of.	−0,22	0,626	0,01	0,956	0,01	0,598
9.	I do not know how healthcare authorities will use the information I provide.	−0,74	0,079	0,09	0,761	0,01	0,687
10.	I cannot report occasionally due to the failure of the informatics system (system unavailable).	−0,11	0,832	−0,01	0,687	0,01	0,671

The strength of association was assessed using General Linear Regression model and expressed in the table by the regression coefficient (Coef.). It is the estimated change in agreement score caused by one unit increase in that particular variable. One unit increase in variable ‘Gender’ is changing from male to female physician, for ‘Age’ and ‘Years of practice’ is one year increase. The probability that the coefficient not different from zero is given under ‘p’. Statistical significant coefficients at 5% level are in bold

**Table 3:** Association between agreement score and practice location

	Statements	City		Smal town		Village	
		Coef.	p	Coef.	p	Coef.	p
1.	The Hungarian infection surveillance system works well.	0,42	0,311	0,53	0,167	0,31	0,938
2.	Severe infectious diseases are detected in special care.	0,39	0,384	0,41	0,332	−0,91	0,841
3.	I only report lab proven infection cases.	−0,72	0,271	−0,34	0,577	**−1,54**	**0,021**
4.	Infection cases exclusively reported by one single doctor do not contribute to epidemiology surveillance.	0,16	0,791	0,66	0,269	0,41	0,522
5.	Reporting infections cases hinders my daily clinical work.	−0,28	0,632	−0,32	0,559	**−1,23**	**0,042**
6.	Reporting infection cases is not only an obligation by law but is also a professional task as well.	0,57	0,071	0,34	0,256	**0,87**	**0,007**
7.	Only relevant and severe infection cases should be reported.	−0,23	0,709	−0,18	0,756	−1,08	0,084
8.	Reporting infections requires more time that we are sometimes short of.	−0,91	0,137	−0,68	0,238	**−1,32**	**0,033**
9.	I do not know how healthcare authorities will use the information I provide.	−0,93	0,103	−0,24	0,654	0,13	0,808
10.	I cannot report occasionally due to the failure of the informatics system (system unavailable).	−0,08	0,903	−1,13	0,079	−0,03	0,961

The strength of association was assessed using General Linear Regression model and expressed in the table by the regression coefficient (Coef.). It is the estimated change in agreement score caused by one unit increase in that particular variable. One unit increase for variable location of practice is the change from capital to ‘City’, or ‘Small town’, or ‘Village’. The probability that the coefficient not different from zero is given under ‘p’. Statistical significant coefficients at 5% level are in bold

From the family physician’s point of view, the epidemiological surveillance system works well (Table 1. Statement 8). However, it should be considered that most of the responders exclusively report only lab proven infections (Table 1. Statement 4).

Family physicians usually consider reporting infected patients as a professional task, though many of them do not have enough time to do it (Table 1. Statement 5 and 10). The majority of them do not know exactly how the transferred and reported data will be used by the healthcare authorities (Table 1. Statement 9). Some physicians believe that a single report cannot contribute to the surveillance systems (Table 1. Statement 1). It was also reported that the electronic reporting system frequently failed to function (Table 1. Statement 3). The majority of physicians agree that only severe infections are reported in special care (Table 1. Statement 6–7).

According to the analyses, three of the four examined demographic characteristics (age, place of practice, years spent in practice) showed significant association with the reporting habits. Older physicians frequently believed that one single report cannot contribute to the operation of the epidemiology surveillance system (Table 2. Statement 4). Physicians, who had spent a longer time in practice, were more likely to consider the epidemiological reporting system as effective (Table 2. Statement 1).

Female physicians were agreed that more severe infectious diseases can be diagnosed at primary care level and also confirmed less influence on their everyday practice (Table 2. Statement 2, 5). They have a higher awareness of epidemiology (Table 2. Statement 9).

The reporting habits were often influenced by the geographic location of the practices. Rural family physicians considered more frequently the reports as a professional task instead of obligation only (Table 3. Statement 6). Those who worked in small villages agreed less reporting only lab proven cases (Table 3. Statement 3). Urban based physicians were hindered less by failures of the computer- system (Table 3. Statement 5, 8).

## Discussion

This is the first Hungarian study providing evidence that certain demographic characteristics of family physicians are related to their reporting habits of infectious diseases. Furthermore, it reflects the different points of view of primary care providers working in different geographical locations. Previous studies indicated that family physicians did not like reporting cases not yet proven by a laboratory tests because it could be professionally awkward [10]. This is also the case in Hungary, particularly in rural areas, that are far from available laboratory services. Authors of studies from New Zealand, USA and Spain mentioned the lack of time as hindrance of reporting, similarly to their Hungarian colleagues [11, 17, 18]. Three different studies also identified lack of time as the main barrier to reporting [16, 19, 20]. Other studies stated that missed reports were explained by patients who refused transferring their personal data, by too much paperwork expected from the doctors or simply forgetting it [17, 18]. Hungarian primary care practitioners do not receive any financial incentives for the notification of infectious diseases. Most policy changes in the area of payment systems are inadequately informed by research [21]. Besides this, several studies identified epidemiological knowledge of the practitioner as the most important determinant of notification [15, 22–25]. No doubt, there is room for improvement in Hungary as well. The lack of clear instructions, inadequate dissemination of guidelines and no assistance with reporting procedures, supervision or feedback are the most important reasons for under reporting [25]. In Hungary, there is no legally binding professional guideline for the management of infections in primary care; hence the regulations could be easily disregarded.

Without appropriate epidemiological knowledge, some physicians could believe that a single report is insufficient for surveillance-operations. Frequent failures of the electronic reporting system were noted. These were only partially explained by simple technical reasons; inexperienced staff members (doctors and nurses) could also been responsible, because the use of computer-systems does not have a long tradition in Hungarian primary care. A study also described the epidemiological awareness of female doctors was higher than that of male physicians [11]. Hungarian female physicians had also better awareness of epidemiology, they confirmed readiness for diagnosing even severe infections in primary care and were less influenced by reporting obligations.

Older physicians believed that a single report does not contribute to the operation of the epidemiology surveillance system. This concept is unacceptable, since every submitted report counts. If health care authorities are unaware of an infected person, they cannot take the necessary preventive actions. Consequently, the infection may be transferred to other individuals, thus increasing the burden on health care providers, as well as governmental administrators.

Moreover, it emphasises the importance of further educational programmes in epidemiology, because it represents only a small part in undergraduate education and vocational training as well [25, 26].

The reporting habit of physicians was often influenced by the geographic location of practices. Rural practitioners were mostly considering the report as not only an obligation by law but also a professional task; consequently they were the most committed. They were less likely to report only lab proven and confirmed cases, certainly due to the restricted availability of microbiology laboratories compared to the capital or larger cities although they were more hindered by IT issues.

We could to reach the appropriate representativeness in the study, covering 8% of all physicians providing primary health care for adults. All of the medical faculties were involved in the research, covering the whole country geographically. The mean age of study population and the national age-data was almost the same.

This research has limitations. The participants involved voluntarily, which may not reflect the entire study population. Furthermore, the survey took place in a specific period of time, and other results could have been obtained in another occasion.

## Conclusion

Our results call for a more systematic control by health care authorities. In addition, general practitioners’ knowledge on epidemiology should be improved, with more updated educational programmes organised by university departments and/or governmental bodies. Inadequate knowledge is leading to unsatisfactory attitude that influences the practices. The epidemiological surveillance and electronic reporting systems should also be technically improved.

## Additional files

Additional file 1: Notifiable infectious diseases. The named infectious diseases’ list is reported according to the Order 18/1998-as (VI. 3.) of the Ministry of Welfare about prevention of infectious diseases and tasks to prevent epidemics. (DOCX 22 kb)

Additional file 2: Survey for family physicians. This questionnaire including demographic questions and 10 statements about the reporting habits of family physicians is related to infectious cases. (DOCX 67 kb)
