# Supplementary material for: An appraisal: how notifiable infectious diseases are reported by Hungarian family physicians
Source: BMC Infect Dis. 2018 Jan 17;18:45. doi: 10.1186/s12879-018-2948-5 (PMC5773032; doi:10.1186/s12879-018-2948-5)
Supplement: Supplementary file 1 — Notifiable infectious diseases. The named infectious diseases’ list is reported according to the Order 18/1998-as (VI. 3.) of the Ministry of Welfare about prevention of infectious diseases and tasks to prevent epidemics. (DOCX 22 kb) [file 12879_2018_2948_MOESM1_ESM.docx]

Appendix I.

**Notifiable infectious diseases**

(Accordong to the Order 18/1998-as (VI. 3.) of the Ministry of Welfare about prevention of infectious diseases and tasks to prevent epidemics [15].)

| *Reported with identifying data* |
| --- |
| Acute flaccid paralysis |
| Amoebiasis |
| Anthrax |
| Botulism |
| Brucellosis |
| Campylobacteriosis |
| Creutzfeldt-Jakob disease |
| Chikungunya |
| Cholera |
| Cryptosporidiosis |
| Diphtheria |
| Echinococcosis |
| Encephalitis infectiosa |
| E. coli enteritis (EHEC, ETEC, EIEC,EPEC, EAggEC,DAEC) |
| Febris flava |
| Giardiasis |
| Haemophilus influenzae |
| Hantavirus |
| Hepatitis infectiosa |
| Hepatitis A |
| Hepatitis B |
| Hepatitis C |
| Hepatitis E |
| Keratoconjunctivitis epidemica |
| Tick borne encephalitis |
| Legionellosis |
| Leptospirosis |
| Listeriosis |
| Lyme disease |
| Lyssa |
| Avian influenza |
| Malaria |
| Malleus |
| Meningitis purulenta |
| Meningitis serosa |
| Meningitis epidemica |
| Morbilli |
| West nile fever |
| Ornithosis |
| Paratyphus |
| Parotitis epidemica |
| Pertussis |
| Plague |
| Poliomyelitis |
| Q-fever |
| Rotavirus gastroenteritis |
| Rubeola |
| Congenital rubeola syndrom |
| Salmonellosis |
| Scarlatina |
| Shigellosis |
| Severe Acute Respiratory Syndrome, (SARS) |
| Streptococcus pneumoniae invasive disease |
| Strongyloidosis |
| Taeniasis |
| Tetanus |
| Toxoplasmosis |
| Tuberculosis |
| Trichinellosis |
| Tularemia |
| Typhus abdominalis |
| Typhus exanthematicus |
| Varicella |
| Variola |
| Virus haemorrhagiac fevers |
| Yersiniosis |
| *Reported without identifying data* |
| AIDS disease |
| HIV-infection |
| Acut urogenital chlamydiasis |
| Gonorrhoea |
| Lymphogranuloma venereum |
| Syphilis |
