# Supplementary material for: An appraisal: how notifiable infectious diseases are reported by Hungarian family physicians
Source: BMC Infect Dis. 2018 Jan 17;18:45. doi: 10.1186/s12879-018-2948-5 (PMC5773032; doi:10.1186/s12879-018-2948-5)
Supplement: Supplementary file 2 — Survey for family physicians. This questionnaire including demographic questions and 10 statements about the reporting habits of family physicians is related to infectious cases. (DOCX 67 kb) [file 12879_2018_2948_MOESM2_ESM.docx]

Appendix II.

**Survey for family physicians**

*Dear Colleague,*

*Reporting infectious diseases belongs to the obligations of family physicians. Please help us improve the Hungarian epidemiology system.*

*Mark or underline your answer.*

Gender Male Female

Birth: 19.. Length of individual practice:………… years

Geography of practice: Budapest, (county center) city, small city, village

0 = I fully disagree

10 = I fully agree


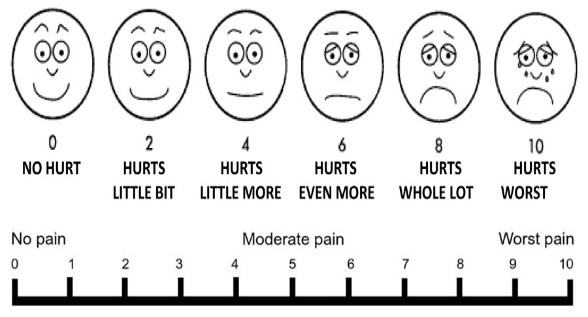


1. The Hungarian infection surveillance system works well.


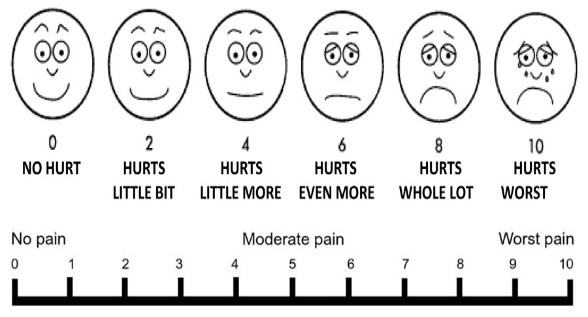


1. Severe infectious disease are detected in special care.
2.
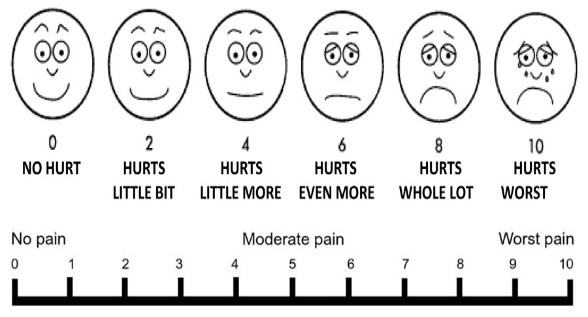
I only report lab proven infection cases.


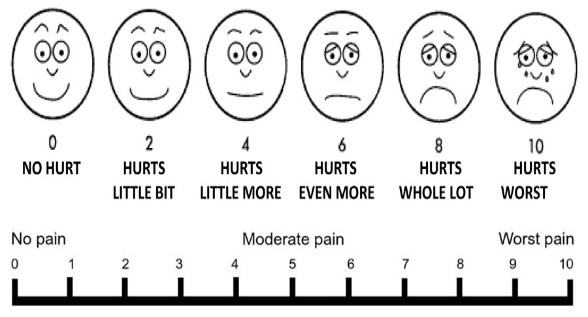


1. Infection cases exclusively reported by one single doctor do not contribute to epidemiology surveillance.


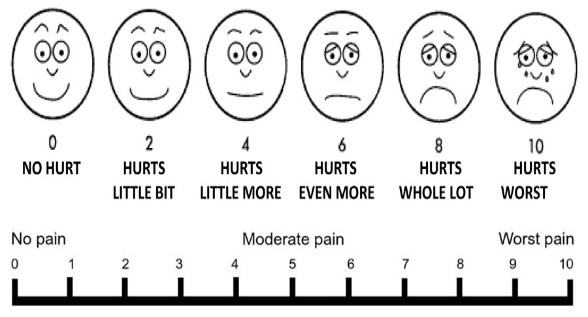


1. Reporting infection cases hinders my daily clinical work.
2.
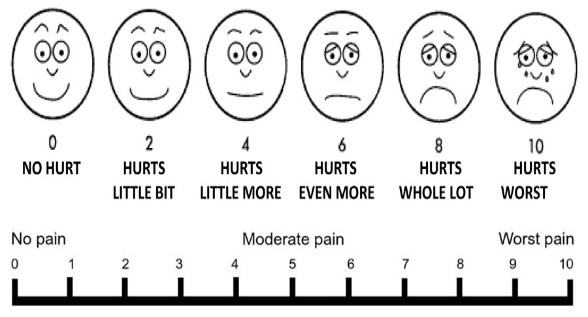
Reporting infection cases is not only an obligation by law but also a professional task.


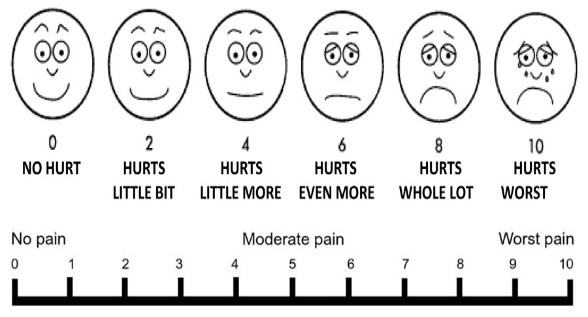


1. Only relevant and severe infection cases should be reported.
2.
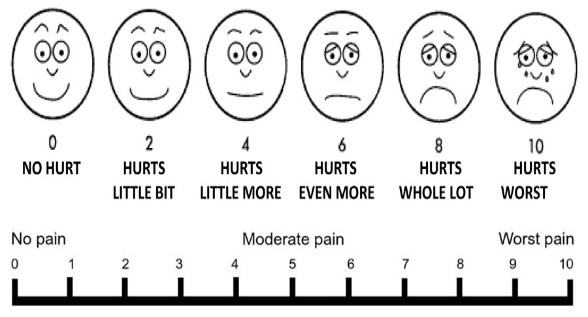
Reporting infections requires more time that we are sometimes short of.


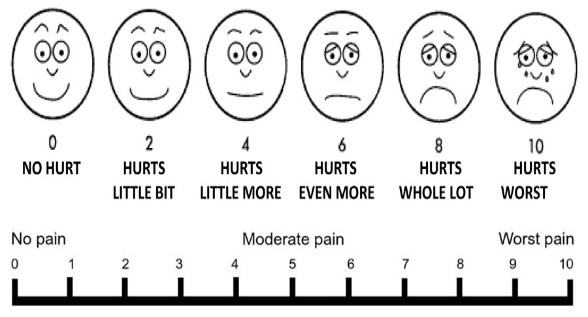


1.
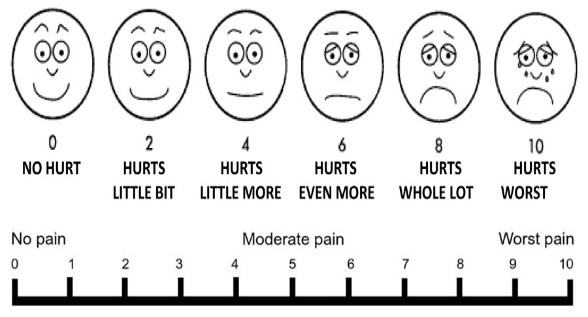
I do not know how healthcare authorities will use the information I provide.
2. I cannot report occasionally due to the failure of the informatics system (system unavailable).
